# Effects of Exercise on Gene Expression of Inflammatory Markers in Human Peripheral Blood Cells: A Systematic Review

**DOI:** 10.1007/s12170-015-0463-4

**Published:** 2015-05-21

**Authors:** Gyrd O. Gjevestad, Kirsten B. Holven, Stine M. Ulven

**Affiliations:** University of Oslo, Oslo, Norway; TINE SA, Oslo, Norway; Norwegian National Advisory Unit on Familial Hypercholesterolemia, Oslo University Hospital, Oslo, Norway; Oslo and Akershus University College of Applied Sciences, Oslo, Norway

**Keywords:** Physical activity, Exercise, Gene expression, Inflammation, Atherosclerosis, Peripheral mononuclear blood cells, PBMCs, Leukocytes, Lymphocytes, Monocytes

## Abstract

Regular physical activity seems to be one of the most important contributors to prevent disease and promote health. Being physically active reduces the risk of developing chronic diseases such as cardiovascular disease, diabetes, and some types of cancers. The molecular mechanisms are however not fully elucidated. Depending on duration and intensity, exercise will cause disruption of muscle fibers triggering a temporary inflammatory response. This response may not only involve the muscle tissue, but also peripheral tissues such as white blood cells, which are important components of the immune system. The immune system plays a vital role in the development of atherosclerosis, thereby making white blood cells relevant to study when looking at molecular mechanisms induced by physical activity. In this review, we summarize the existing literature on exercise and gene expression in human white blood cells, and discuss these results in relation to inflammation and atherosclerosis.

## Introduction

There are substantial epidemiological evidence that regular physical activity (exercise) reduce the risk of developing diseases such as cardiovascular disease [1–4], type 2 diabetes [2, 4, 5], and some types of cancers [2, 4, 6]. Regular physical activity is therefore one of the most important contributors to maintaining health. The mechanisms by which exercise contributes to health are however not fully understood.

An acute bout of exercise, depending on type, intensity, and duration [7–9], causes tissue injury, triggering a local and systemic inflammation with a release of both pro- and anti-inflammatory cytokines [10, 11], while regularly physical activity seems to attenuate the inflammatory response promoting an anti-inflammatory environment in the body [3, 12, 13].

The inflammatory response may be studied through changes in circulating biomarkers, such as interleukins, chemokines, and other signaling molecules. When studying possible effects of exercise in relation to atherosclerosis, biomarkers for underlying processes, such as endothelial dysfunction, oxidative stress, and inflammation are relevant (Table 1).

**Table 1:** Common inflammatory markers, included in this review, and their biological functions relevant for atherosclerosis and physical activity

Inflammatory markers	Gene symbol	Function
Chemokine (C-C motif) ligand 2	CCL2	Involved in chemotactic activity for monocytes and basophils, binding to CCR2 and CCR4.
Chemokine (C-C motif) ligand 3	CCL3	Involved in the acute inflammation by recruitment and activation of leukocytes.
Chemokine (C-C motif) ligand 4	CCL4	Involved in the migration of immune cells, a chemoattractant.
Chemokine (C-C motif) ligand 5	CCL5	Involved in recruiting leukocytes to inflammatory sites.
Chemokine (C-C motif) receptors (2, 3 and 4)	CCR2, 3, 4	Involved in the regulation of cell trafficking, important in inflammation, binds to cytokines.
Chemokine (C-X-C motif) ligand 16	CXCL16	Involved in the migration of immune cells, a chemoattractant.
Endothelial nitric oxide synthase	NOS3	Involved in the generation of NO in blood vessels, regulating vascular tone, and platelet aggregation.
GATA binding protein 3	GATA3	T cell-specific transcription factor involved in the regulation of T cell development.
Glutathione peroxidase	GPX	Involved in the detoxification of hydrogen peroxide.
Heat shock 27 kDa protein-associated protein 1	HSPBAP1	Involved in stress resistance; actin organization, and translocation from the cytoplasm to the nucleus.
Heat shock 70 kDa protein 1A	HSPA1A	Involved in stress resistance; stabilizing proteins against aggregation and mediates the folding of newly translated proteins.
Heat shock 70 kDa protein 6	HSPA6	Involved in stress resistance; protein folding, stabilization, and shuttling functions in response to stress.
Inducible nitric oxide synthase	NOS2	Involved in immune response and important in cellular signaling, produces NO.
Interferon gamma	IFNG	Involved in the regulation of immune and inflammatory response, promotes Th1 differentiation.
Interleukin 1 receptor antagonist	IL1RN	Inhibits the activity of IL1A/IL1B, and modulates a variety of interleukin 1-related immune and inflammatory responses.
Interleukin 1 receptor-like 1	IL1R1	Involved in cytokine-induced immune and inflammatory response.
Interleukin 10	IL10	Downregulates the expression of Th1 cytokines, enhances B cell survival, proliferation, and antibody production, able to block NF-κB activity.
Interleukin 13	IL13	Immunoregulatory cytokine that plays a role in B cell maturation and differentiation, downregulates macrophage activity inhibiting the production of pro-inflammatory cytokines and chemokines.
Interleukin 1a, interleukin 1b	IL1A/IL1B	Proliferation and maturation of lymphocytes, involved in inflammation and acute-phase response.
Interleukin 4	IL4	Pleiotropic cytokine involved in T cell and macrophage differentiation and modulate the differentiation to Th2.
Interleukin 6	IL6	A pleiotropic cytokine that plays important roles in inflammation and the acute-phase response.
Interleukin 8	IL8	Involved in the acute inflammatory response, a chemoattractant.
Matrix metallopeptidase 9	MMP9	Involved in the breakdown of extracellular matrix and tissue remodeling.
NADPH oxidase	NADPH oxidase	Involved in the vascular superoxide production.
Prostaglandin-endoperoxide synthase 2	PTGS2	An enzyme responsible for formation of prostanoids, involved in inflammation.
Superoxide dismutase 1	SOD1	Involved in the anti-oxidative defense destroying free superoxide radicals in the body.
Superoxide dismutase 2	SOD2	Involved in the anti-oxidative defense destroying free superoxide radicals in the body.
TNF receptor-associated factor 6	TRAF6	Involved in signal transducing in NF-kappa B pathway.
Toll-like receptors 2, 4, and 7	TLR2, 4, and 7	Involved in recognition of pathogen-associated molecular patterns (PAMPs), mediate the production of cytokines necessary for the development of effective immunity.
Transforming growth factor beta	TGFB	Involved in proliferation, differentiation, adhesion, and migration.
Tumor necrosis factor alpha	TNF	Prototypical pro-inflammatory cytokine, plays a central role in inflammation, immune system development, and apoptosis.

Peripheral white blood cells are important components of the immune system, and the immune system is important in the development of atherosclerosis [14]. White blood cells are constantly interacting with other cells, such as endothelial cells in the arteries [15–17], making them relevant for studying the inflammatory process in atherosclerosis [17–19].

It is vital for the body to regulate the expression of genes in the process of adapting to changes in the environment, such as exercise [20]. Gene expression studies can be used to get an insight into molecular mechanisms in affected cells, and to reflect the early stages of activation in the immune system. Gene expression studies may therefore be a sensitive tool to characterize the early effects of exercise on immune regulation [12].

We have summarized gene expression studies in human white blood cells, including peripheral mononuclear blood cells (PBMCs), lymphocytes and monocytes, but excluding natural killer (NK) cells. Studies, both acute exercise interventions and interventions studies investigating the prolonged effects of exercise, have been included.

## Literature Search

A literature search was conducted in a combined search in Ovid Medline and EMBASE in November 2014 and re-ran in February 2015. Medical subject headings (MeSH) combined with words, some of them truncated, in title or abstract (tw) or only in title (ti), was used as follows: leukocytes, mononuclear/ or exp lymphocyte subsets/ or exp b-lymphocytes/ or exp t-lymphocytes/ or monocytes/ or (leukocyte* or mononuclear or monocytes or lymphocyte* or macrophage*).tw. AND exp exercise/ or exp physical endurance/ or (exercise* or physical activit* or athlete*).tw. or training.ti. or Athletes/ AND gene expression/ or exp transcription, genetic/ or gene expression regulation/ or down-regulation/ or gene amplification/ or protein modification, translational/ or protein processing, post-translational/ or rna processing, post-transcriptional/ or transcriptional activation/ or up- regulation/ OR (gene expression or transcriptom or rna or mrna).tw. exp RNA AND exp Cytokines/ or exp Inflammation/ or exp Immune System Phenomena/ or (cytokine* or interleukin* or inflammat* or immun*).tw.

The search was limited to humans with: not animals (including studies were data on humans and animals were reported separately) and to articles published in English, Danish, Norwegian, or Swedish.

**Fig. 1:**
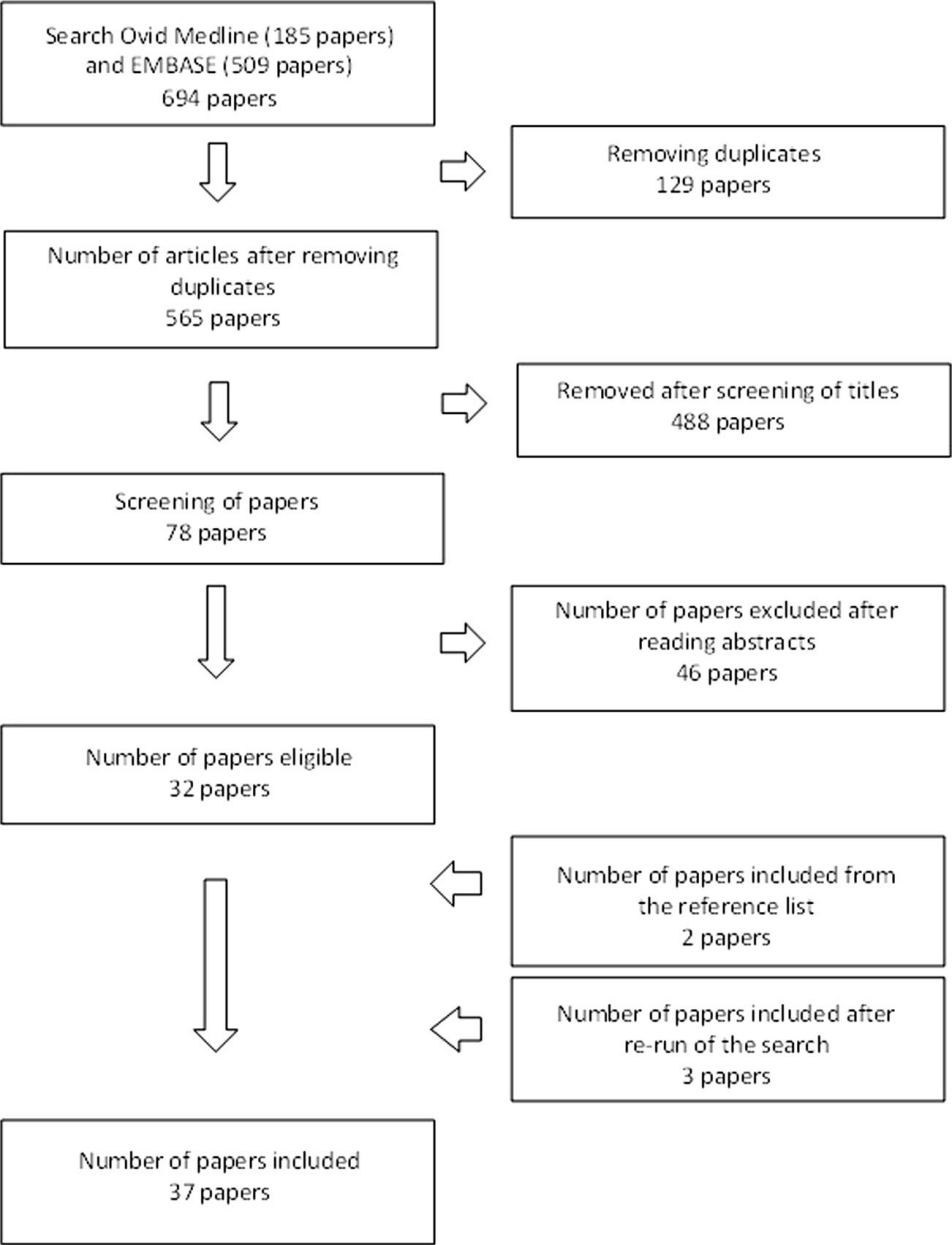
Flow chart showing the result of the combined literature search in Ovid Medline and EMBASE and the selection of papers

After removing duplicates, 565 papers were identified. Only original papers and papers including invention studies with leukocytes, including PBMCs, monocytes, lymphocytes, and dendrite cells, were included. Studies were excluded if only whole blood was analyzed, no gene expression data were presented, or the intervention included giving food supplements in combination with exercise. Studies including NK cells only were not included. Neither were papers including subjects on a weight reduction program. Using these criteria, the number of relevant articles was 78. Another 46 papers were excluded after reading the abstract/full article. Additional two articles, identified from the reference lists of the already included papers, were found to be relevant and included in the review. After a re-run of the search, three new articles were included in the review. In total, 37 papers are included in the review as shown in Fig. 1.

## Acute Exercise and Gene Expression Studies

Table 2a summarizes the effect of an acute bout of exercise on gene expression in white blood cells from 28 studies. In 26 of the studies, the subjects performed an acute bout of endurance exercise, while in the remaining two studies subjects performed an acute bout of strength exercise. In 18 of the studies, PBMCs were investigated, while in the other studies leukocytes (seven studies), lymphocytes (two studies), and monocytes (one study) were investigated.

**Table 2:** Gene expression studies performed in human white blood cells (lymphocytes, monocytes, and PBMCs) after exercise

Study	Intervention	Description of exercise	Subjects (age, n, gender)	Genes investigated	Regulation after exercise (↑,↓, ↔)
(a) Gene expression studies performed in white blood cells after an acute bout of exercise					
Ullum et al. [21]	Moderately trained subjects cycled for 1 h. Blood samples were drawn before exercise, during the last minute of exercise, and 2 and 4 h after exercise. For six subjects, blood samples were also drawn 1, 3, 5, and 6 h after exercise.	Ergometric bicycle exercise for 1 h at 75 % of VO_2_max.	*n* = 17 29–39 years male	IL6, IL1A, IL1B, TNF	IL1A, IL1B, IL6, and TNF ↔
Natelson et al. [22]	Walking on a treadmill until exhaustion. Blood samples were taken before exercise and 10 min into recovery.	Gradually increasing walking speed up to 6.4 km/h. Thereafter, the speed was held constant, but the incline was increased every min with 2 % grades. Subjects stopped walking at exhaustion (45.6 ± 0.8 min).	*n* = 7 39.3 ± 4.7 years male (disabled, but healthy)	TNF, IL1A, IL1B, IL2, IL4, IL10, and IFNG	TNF ↓, IL1A, IL1B, IL2, IL4,IL10, and IFNG ↔
Ostrowski et al. [23]	Subjects participated in a marathon race. Blood samples were taken 1 week before, immediately after exercise, and 2 h into recovery of the marathon race.	Marathon race	*n* = 16 30.5 ± 1.9 years male	IL6, IL1RN, IL1B, and TNF	IL1RN↑ in 5 PBMC samples. IL1B↑ in 4 PBMC samples. IL6 and TNF-α not detected.
Fehrenbach et al. [24]	Two groups: trained athletes (*n* = 12) and untrained controls (*n* = 12). The trained group performed a half-marathon. Blood samples were drawn from both groups 24 h before the race, immediately after exercise, and 3 and 24 h into recovery.	Trained athletes (53.3 ± 18.4 km/week) performed a half-marathon. Untrained subjects were sitting in the laboratory.	*n* = 24 trained 32.3 ± 9.3 years untrained 45.4 ± 11.4 years male	HSPBAP1, HSPA6	HSPBAP1 and HSPA6↑ in athletes, returned to baseline after 24 h in athletes.
Moldoveanu et al. [25]	Untrained subjects exercising 3 h at 60–65 % VO_2_max. Blood samples were taken at baseline and 30, 60, 120, 180, 210, 240, and 300 min after baseline. Subjects came in for a final blood sample 24 h after baseline. Subjects performed both an exercise and a control trial at corresponding periods of the day with at least 7 days apart.	1 h cycling, 1 h inclined treadmill, and 1 h cycling without pause or recuperation.	*n* = 10 25 ± 5 years male	IL6, IL1B, and TNF	IL6, IL1B, and TNF ↔
Niess et al. [26]	Well-trained athletes (55.7 ± 5.5 km/week) and untrained subjects (<3 h recreational activity/week) were included. The athletes (TG) ran a half-marathon, while the untrained group (CG) performed an exercise test on a treadmill. Blood samples were collected before exercise; immediately after; 3, 24, and 48 h into recovery.	Half-marathon and graded exercise test. Graded exercise test on a treadmill—speed was increased with 2 km/h every third minute until exhaustion. The incline was kept constant at 1 %. After 15 min rest, a continuous ran was performed at 110 % of the anaerobic threshold until exhaustion.	n TG = 10 n CG = 8 trained 32.3 ± 3.3 years untrained 25.0 ± 2.2 years male	NOS2	NOS2 ↑ directly after the half-marathon (peak after 3 h). ↔ NOS2 after the continuous run in untrained subjects.
Thompson et al. [27]	Habitually active (5 ± 1 h per week) subjects performed an exercise and a rest trial (sitting calmly in the lab). Blood samples were collected before, immediately after exercise, and 1 and 2 h into recovery. A final blood sample was collected the following day.	75 min of running on a treadmill at 70 % VO_2_max.	*n* = 8 21 ± 1 year male	HO-1	HO-1↑, the peak varied in time among the individuals. HO-1 ↔ in control trial (rest)
Ferrer et al. [28]	Swimmers on an amateur team participated in a 1 h swimming session.	A series of intermitted 50 m swim of progressively increasing speed for 30 min, with a 10–15 s rest between swims. The next half hour, the swimmers continued swimming 50 m, with 10–15 s rest, at 75–80 % of VO_2_max.	*n* = 15 boys *n* = 9 girls boys 16.1 ± 0.5 years girls 14.7 ± 0.2 years male and female	Catalase, GPX, BCL2, PPARGC1A, and UCP-3	BCL2, UCP-3 ↓ for both genders, catalase, GPX, PPARGC1A, ↔ in both genders.
Sakharov et al. [29]	Trained skiers performed a treadmill test; 15 ± 0.5 min. Blood samples were taken before and immediately after exercise.	Step-by-step increasing power treadmill test; initial treadmill velocity 3.0 m/s, slope angle 1°, velocity increment 0.5 m/s.	*n* = 4 19.3 ± 0.7 years gender not specified	HSPA1A	HSPA1A↑
Sureda et al. [30]	Soccer players played a training match. Blood samples were taken before and after the match.	60-min soccer game with different intensities (low 70–80 %, medium 80–90 %, and high 90–100 %).	*n* = 18 low 20.2 ± 0.4 years medium 19.8 ± 0.3 years high 19.7 ± 0.4 years male	HO-1	HO-1↑ in moderate and high intensity groups. OH-1 ↔ between groups post-exercise.
Jenkins et al. [31]	Endurance-trained athletes (*n* = 10) performing at least 4 h/week of endurance training, and sedentary people (*n* = 10) engaging in exercise <20 min/day on 2 days/week, ran 30 min on a treadmill. Blood samples were taken before and after the test.	30-min treadmill running at 75 % VO_2_max.	*n* = 20 25 ± 1 year male	NOS3, SOD, SOD1, SOD2, GPX1, CD34, and VEGF	In CD34+ cells: SOD1↓ in sedentary group after exercise. NCF1 and NOX2↓ in both groups. VEGF, SOD2, GPX1 between groups ↔ VEGF. In CD34- cells: SOD1 and SOD2↑ in the sedentary group. NOS3↑ in sedentary compared to trained. VEGF, NOS2, GPX1, SOD1, SOD2, NCF1, and NOX2 between groups ↔
Li et al. [32]	Healthy tai chi (TC) players performed a Yang style TC. Blood samples were collected before and immediately after the exercise.	1 h Yang style TC consisting average 5 min warm-up, TC for 45 min, and 5 min cooldown.	*n* = 3 50–90 years gender not specified	IL13	IL13↑
Nickel et al. [33]	Three age-matched groups depending upon exercise level; lean elite (*n* = 16, LE, regularly exercising, ≥55 km/week), lean non-elite (*n* = 16, LNE) and obese non-elite (*n* = 15, ONE)—two lasts groups; ≤40 km/week only pre-marathon exercise. Fasting blood samples were taken 5–2 days before the race, immediately, and 24 h after the marathon.	Marathon race	*n* = 47 ONE 40 ± 6 years, LNE 40 ± 6 years, LE 40 ± 7 years male	TLR2, TLR4, TLR7	Right after the marathon: LNE: TLR4 ↓ LE, ONE: TLR4 ↔ All groups: TLR7 ↓ 24 h after the marathon: All groups: TLR4 and TLR7↑ TLR2 ↔ between groups or before/after marathon.
Thomas et al. [34]	Untrained subjects were recruited to an acute bout of exercise. Blood samples were collected before; immediately after exercise; and 1.5, 3, and 24 h into recovery.	45-min cycling at 70 % of VO_2_max.	*n* = 9 32 ± 8 years gender not specified	PPARG, CD36, NR1H3, ABCA1, PPARGC1A, CETP, L-CAT, APOA1	CD36, NR1H3, ABCA1↑ PPARG, PPARGC1A, CETP, and L-CAT ↔
Bernecker et al. [35]	Participants participated in a marathon race. Blood samples taken directly before and within 1 h after finishing the race.	Marathon race	*n* = 13 43.0 ± 10.9 years male	TNF, IL6	TNF, IL6 ↔
Ulven et al. [36]	Well-trained subjects performed a 1-h cycling. Blood samples were drawn before and after exercise. The exercise test day was repeated twice.	One-hour ergometer cycling at 70 % of VO_2_max.	*n* = 10 25 (22–28) yrs male	18 genes	IL1B, CXCL16, IL8, PTGS2, TBX21, and GATA3↑ TLR2↓ TNF, CD40, CD40L, TGFB, IFNG, IL18, TLR4, TLR6, CD3E, CD8A, and FOXP3 ↔
Xiang et al. [37]	Trained subjects were randomly recruited from a lager cohort to run a marathon race. Average training mileage before the race was 17.4 ± 9.1 miles/week. Blood samples were collected 24–48 h before and 1 week after completing the race.	Marathon race	*n* = 16 41.0 ± 2.6 years female (5), male (11)	84 genes	IL4, GATA3, CCR4, CCR3, IRF1, CCR2, CEBPB, GPR44, NFATC2, NFATC2IP, TMED1, LAG3, LAT, MAP2K7, CD28, CD8 ↑ IFNG/IL4 ratio, and TBX21/GATA3 ratio↓ Th3-related gene expression pattern ↔
Connolly et al. [38]	Healthy subjects were included to perform a cycle ergometric. Blood samples taken before exercise, end of exercise, and 60 min into recovery.	30-min constant-work-rate ergometric cycling (80 % peak VO_2_max)	*n* = 15 25.2 ± 0.8 years male	Whole genome	311 genes diff reg. between pre- and end-ex, such as HSPA1A, HSPA1B, CCL3, CCL4, CLL5, NR4A2 and RGS1↑, IL1RN, and CD14 ↔ 552 genes diff reg. between end-ex and recovery, such as IL6R, IL1RN, CD14↑, CCL3, CCL5, and NR4A2↓ 292 genes diff reg. between pre- and recovery, such as CD14↑, CCL3, CCL4, CCL5, IL1RN, NR4A, IL6, IL10, and soluble TNF receptor ↔
Buttner et al. [39]	Subjects already participating in leisure activities (6.0 ± 2.6 h/week) performed a strenuous treadmill exercise. Two weeks after they performed a moderate treadmill exercise. Blood samples were taken before and 1 h after exercise.	One bout of strenuous treadmill exercise (80 % of VO_2_max until exhaustion—mean time 39.0 ± 14.8 min) and one bout of moderate treadmill exercise (60 % of VO_2_max), identical time periods.	*n* = 5 25.4 ± 3.5 years male	Whole genome	39 genes↑, among these HSPA1A, MMP9, IL8RA, IL1 receptor, SLC2A3, and IL1R2, 7 genes↓, among these YES and CD160
Radom-Aizik et al. [12]	Early- and late puberty girls (not involved in competitive sports) performed ergometric cycling. Blood were obtained at rest and after exercise.	Ten 2-min bouts of constant-work-rate ergometric cycling with 1-min rest between each interval. The work rate was individualized to 50 % of VO_2_max.	*n* = 10 (both groups) 10.0 ± 0.3 years 16.1 ± 0.4 years females	Whole genome	Late puberty; 611 genes ↑, 266 genes ↓ Early puberty; 829 genes ↑, 491 genes ↓ 622 genes were commonly regulated in both groups (420↑, 202 ↓), 255 genes were differently expressed in late puberty, but not in early puberty. 698 were expressed in early puberty, but not in late puberty.
Radom-Aizik et al. [12]	Early- and late puberty boys (not involved in competitive sports) performed ergometric cycling. Blood were obtained at rest and after exercise.	Ten 2-min bouts of constant-work-rate ergometric cycling with 1 min rest between each interval. The work rate was individualized to 50 % of VO_2_max.	*n* = 10 (both groups) 10.5 ± 0.4 years 17.4 ± 0.4 years male	Whole genome	Late puberty; 517 genes (CCL4, FASLG, GZMA, PRF1, and HAPA1B) ↑ and 729 genes (IL8)↓ Early puberty; 79 genes (FASLG, HAPA1B) ↑ and 30 genes ↓ 66 genes were commonly regulated in both groups, 64 of which were regulated in the same direction. For 37 of the common upregulated genes, the average fold change was the same in the two groups. The same was the case for 27 common downregulated genes.
Carlson et al. [40]	Trained subjects with weight lifting experience performed an acute bout of resistance exercise—30 min following a 12-h fast. Blood samples were taken at rest (baseline), immediately after exercise (post-ex), and 2 h into recovery.	Six sets of parallel back squat followed by six sets of seated leg press. Each exercise consisted of two warm-up sets of 10 repetitions at 45 and 55 % of 1 RM and four sets of 10 repetitions of 65 % of 1 RM. 2-min resting period between sets were allowed.	*n* = 10 22.3 ± 1.3 years male	Whole genome	From baseline to post-ex (six genes); NR4A2, CREM, EREG, AREG, DUSP2, and RGS1 ↑. From post-ex to recovery (259 genes): MMP9, DAAM2, ORM1, ARG1↑, DUSP2, NR4A2, XCL1, PDGFD, SIK1, and AREG↓ From baseline to recovery (167 genes): MMP9, ORM1, DAAM2, CD160, ARG1, TPST1↑, DUSP2, CCL4, LAIR2, XCL1, PDGFD, CD160, and XCL1-markers of lymphocyte population ↓.
Kimsa et al. [41]	Ergometric cycling looking at the expression pattern of TGFB-signaling pathways. Blood samples were drawn before exercise (pre-ex), immediately after exercise (post-ex), and 15 min into recovery.	Unloaded cycling for 5 min, intensity increased by 40 W every 3 min up to maximal exercise intensity and 60–70 rpm was maintained.	*n* = 3 26.7 ± 7.8 years male	Whole genome	Pre-ex to post-ex: RUNX3, TGFBR3, and MLC1↑ Pre-ex to 15-min recovery: GRB2↑, RUNX3, and TGFBR3↓
Maltseva et al. [42]	Skiers engaged in regular training for the last 5 years were included for a treadmill run. Blood samples were taken before, directly after exercise, and 30 and 60 min into recovery.	Treadmill running for 30 min at 80 % of VO_2_max.	*n* = 9 19.3 ± 0.7 years gender not specified	Whole genome	HSPAIA↑ after exercise, stabilized during recovery. PGLYRP1↑ after exercise and continued to increase throughout recovery.
Sakharov et al. [43]	Highly trained skiers participated in a treadmill test (RTE). Two weeks later, seven of these performed a moderate treadmill test (MT) for 30 min. Blood samples were taken before and immediately after both tests.	The initial treadmill test (RTE) was performed until exhaustion with an incremental step protocol. The second treadmill test (MT) was performed at moderate intensity at 80 % VO_2_max for 30 min.	n (RTE) = 19 n (MT) = 7 RTE 20.9 ± 3.2 years MT 22.0 ± 3.7 years male	Whole genome	310 genes ↑ after RTE. 69 ↑ after MT (of which 64 were identical to RTE) Pathways regulated were related to inflammation, stress response, signal transduction, and apoptosis.
Kimsa et al. [44]	Ergometric cycling and expression pattern of inflammation-related genes. Blood samples were drawn before exercise (pre-ex), immediately after exercise (post-ex), and 15 min into recovery.	Unloaded cycling for 5 min, intensity increased by 40 W every 3 min up to maximal exercise intensity and 60–70 rpm was maintained.	*n* = 3 26.7 ± 7.8 years male	Whole genome	Pre-ex to post-ex: IL2RB, IL18R1, TXLNA↑, IL5RA, IL6 ↓ Pre-ex to 15-min recovery: IL1B, IL8, IL18R1↑, IL1R1, CSF2, and TXLNA ↓ Post-ex to 15-min recovery: IL5RA↑, IL2RB, and TXLNA ↓
Radom-Aizik et al. [45]	Participants performed an intermittent-exercise protocol. Blood samples were taken 30 min before exercise (baseline) and immediately after exercise.	Ten 2-min bouts of constant-work-rate ergometric cycling with 1 min rest between each interval. The work rate was individualized to 82 % VO_2_max.	*n* = 12 26.0 ± 0.6 years male	Whole genome	The exercise protocol altered the expression level of 894 annotated genes in the circulating monocytes, such as EREG, CXCR4↑, TNF, TLR4, and CD36↓
Storey et al. [46]	Competitive weight lifters performed a standardized weightlifting program after a period with either intensified or reduced training programs. Blood samples were collected before training, right after exercise, and 3 h into recovery.	Six to eight sets of one to three repetitions of power snatch, power clean, and back squat. Two final sets at 90 % of max. Duration of acute bout, 90 min.	*n* = 7 22.9 ± 4.3 years male (*n* = 4) and female (*n* = 3)	Whole genome and CCL4, CXCR4, and DDIT4	202 regulated genes associated with cell-to-cell signaling and immune cell trafficking, organismal survival, inflammation and cell cycle, and cell death. CCL4, CXCR4 ↑ after exercise in those who had been on an intensified training before the acute bout. DDIT4 ↑ in both groups after exercise.
(b) Gene expression studies performed in white blood cells after prolonged exercise.					
Jimenez-Jimenes et al. [47]	Two bouts of exercise before and after 8 weeks of leg press eccentric training. Training was performed twice a week. Blood samples were taken at rest (baseline), immediately after exercise, and 3 h into recovery.	Eccentric bouts: 10 sets of 10 repetitions. 3-min rest between sets. 60 % of MIVC.	*n* = 11 70.6 ± 4.0 year male	NOS2, PTGS2, IL6	Following the first bout and maintained after 3 h: NOS2, PTGS2, and IL6 mRNA↑. NF-κB activation↑. Following the second bout: IL6 mRNA↔ and maintained after 3 h: NOS2, PTGS2 mRNA ↑ Changes were attenuated following the second exercise bout for NOS2 and PTGS2.
Yakeu et al. [48]	Sedentary subjects—8 weeks low-intensity training program. Blood samples were collected before the intervention and 24 h after the last exercise at the end of intervention.	Walking 10,000 steps/day on a treadmill (within 75 min), three times a week.	*n* = 17 45.6 ± 11.1 year female (*n* = 8) and male (*n* = 9)	CCL2, IL6, IL4, IL10, TNF, MR, CD14, AMAC1, CXCL2, PPARGC1A, PPARGC1B, PPARA, and PPARG/D	PPARGC1A, PPARGC1B, IL4, CD14, and MR↑ IL6, CXCL2, TNF, and CCL2↓
Gano et al. [16]	Walking every day for 2 months. Blood samples taken before and after the intervention period.	40–45 min at 70–75 % VO_2_max.	*n* = 11 57–70 year male (*n* = 5) and female (*n* = 6)	AGER, NCF1, CCL2, NOS2, NF-κB, TNF, and IL6	AGER, NCF1, and CCL2 ↓ NOS2, TNF, and IL6 ↔
Fernandez-Gonzalo et al. [49]	Sedentary people were divided into a training group (TG) and a control group (CG). Both groups performed two acute bouts of exercise, before and after a 6-week eccentric training program. Blood samples were taken before exercise, immediately after, and 2 h into recovery.	Acute bout: 12 sets of 10 repetitions, 60 % of the MVIC, 3-min rest between each set, on a barbell squat. Exercise program: 3 sessions/week, 3–5 sets of 10 repetitions, 40–50 % of MVIC.	n TG = 12 n CG = 8 22.4 ± 0.5 years male	CD14, TLR4, and TNF	First bout of exercise in both groups: CD14, TLR4, and TNF↑ (no diff between groups). Second bout of exercise in both groups: CD14, TLR4, TNF↑ only in CG. CD14 and TLR4 ↔ between groups.
Fernandez-Gonzalo et al. [50]	Sedentary people were divided into a training group (TG) and a control group (CG). Both groups performed two acute bouts of exercise, before and after a 6-week eccentric training program. Blood samples were taken before exercise, immediately after, and 2 h into recovery.	Acute bout: 12 sets of 10 repetitions, 60 % of the MVIC, 3-min rest between each set, on a barbell squat. Exercise program: 3 sessions/week, 3–5 sets of 10 repetitions with loads ranging between 40 and 50 % of MVIC.	n TG = 12 n CG = 8 TG: 22.5 ± 0.3 years CG: 22.5 ± 2.3 years female	CD14, TLR4, and TRAF6	First bout of exercise in both groups: CD14, TLR4, and TRAF↑ Second bout of exercise in both groups: CD14, TLR4, TRAF6↑ but less in TG.
Rodriguez-Miguelez et al. [51]	Two groups; training group (TG) and control group (CG). Resistance training for 8 weeks two times per week, no changes in daily routines for CG. Blood samples were collected before and after the exercise period.	10 min warm-up at a cycle ergometer, three different exercises: leg press, biceps curl, and pec deck. Number of sets 3 × 8, 3 × 10, and 3 × 12 at 60 % of 1 RM during weeks 1–3. 3 × 8, 3 × 10, and 3 × 12 at 70 % of 1 RM during weeks 4–6. 3 × 8, 3 × 10 at 80 % of 1 RM during weeks 7 and 8.	n TG =16 n CG =10 TG 69.1 ± 1.1 year CG 70.0 ± 0.9 years male (*n* = 7) and female (*n* = 19)	IL10, TNF	IL10 ↑ in the TG. TNF ↔ in any groups.
Tringali et al. [52]	Two groups: elite gymnastics and artistic gymnasts (recreational level). mRNA measured at rest in spring and fall. The elite athletes were divided into two groups—before and after reaching menarche.	Elite gymnasts performed 3-h daily training, 18 ± 4 h training a week. Recreational gymnasts performed 2-h training per day, 4.7 ± 0.5 h a week.	*n* = 32 (16 + 16) elite gymnasts; 11.3 ± 1.9 years recreational gymnasts; 13.1 ± 1.5 years female	IL6, IL10, TNF, and IFNG	Elite gymnasts vs. recreational gymnasts; IL6 and TNF ↑in elite gymnasts, IL10 and IFNG ↔ Higher ratio IL6/IL10 and TNF/IL10 in elite gymnasts. IL6↑ in pre-pubertal girls. Pre- vs post-pubertal girls: IL10, TNF, and IFNG ↔
Thompson et al. [15]	24 weeks of training followed by 2 weeks of detraining (removal of exercise). Samples from six individuals with high basal plasma IL6 levels were collected at baseline, at end of exercise, and after 2 weeks of detraining.	Training 3–4 times a week, 30–60 min each time, starting at 50 % of VO_2_max increasing steadily to 70 % of VO_2_max.	*n* = 6 54 ± 5 years male	Whole genome	31 probes returned to baseline after 2 weeks of detraining. 22 probes at the same levels as after 2 weeks of detraining (compared to end of the 24-week training program). After detraining: CLC, PARD6G, IL2, IL8, INSL6, and RET ↑ CPA2, CPS1, and SGOL1 ↓
Dias et al. [53]	Healthy untrained subjects were included to 18 weeks of running three times a week.	80-min session including 5 min warm-up, 60 min run, and 15 min cooldown activities. The intensity corresponded to the anaerobic threshold and respiratory compensation point.	*n* = 13 25 ± 3 years male	Whole genome	152 transcripts↑, 59 transcripts ↓ Genes related to immune function, cell cycle processes, development, and growth.

Studies performed using an acute bout of exercise mostly used PBMCs except for the study of Buttner el al, Fehrenbach et al., Maltseva et al., Nieman et al., Niess et al., Sakharov et al. (two studies) were leukocytes were used and the study of Ferrer et al. and Thompson et al. were lymphocytes were used. In eight out of the nine studies performed using prolonged exercise as the intervention, PBMCs were used. Yakeu et al., 2010 used leukocytes. *↑* indicates genes being upregulated, *↓* indicates genes being downregulated, and *↔* indicates no changes in genes expression after exercise

*MIVC* maximal isometric voluntary contraction, *VO*_*2*_ *max* maximal oxygen uptake, *yrs* years, *h* hours, *IL* interleukin, *IL1B* interleukin 1 beta, *TNF* tumor necrosis factor-α, *IFNG* interferon gamma, *IL1RN* interleukin-1 receptor antagonist, *HSP* heat shock protein, *NOS2* inducible nitric oxide synthase 2, *mRNA* messenger ribonucleic acid, *HO*-*1* heme oxygenase 1, *GPX* glutathione peroxidase, *BCL2* B cell lymphoma 2, *UCP*-*3* mitochondrial uncoupling protein 3, *HSPA6* heat shock protein 70 kDa protein 6, *HSPBAP1* heat shock protein 27 kDa-associated protein 1, *HSPA1A* heat shock 70 kDa protein 1, *NOS3* endothelial nitric oxide synthase, *SOD* superoxide dismutase, *CD* cluster of differentiation, *TLR* toll-like receptor, *PPARG* peroxisome proliferator-activated receptor gamma, *NR1H3* nuclear receptor family 1 group H member 3, *ABCA1* ATP-binding cassette transporter 1, *CEPT* cholesteryl ester transfer protein, *L*-*CAT* phosphatidylcholine-sterol acyltransferase, *ApoA1* apolipoprotein A-I, *CXCL* chemokine (C-X-C motif) ligand, *PTGS2* prostaglandin-endoperoxidase synthase 2, *TBX21* T-box transcription factor 21, *GATA3* trans-acting T cell-specific transcription factor, *TGFB* transforming growth factor-β, *FOXP3* forkhead box P3, *CCR* C-C chemokine receptor, *IRF1* interferon regulatory factor 1, *CEBPB* CCAAT/enhancer-binding protein beta, *GPR44* G protein-coupled receptor 44, *NFATC2* nuclear factor of activated T cells cytoplasmic 2, *NFATC2IP* nuclear factor of activated T cells cytoplasmic 2-interacting protein, *TMED1* transmembrane emp24 domain-containing protein 1, *LAG3* lymphocyte-activation gene 3, *LAT* linker for activation of T cells, *MAP2K7* mitogen-activated protein kinase kinase 7, *TBX21* T cell-specific T-box transcription factor T-bet, *Th* T helper cell, *NR4A2* nuclear receptor subfamily 4 group A member 2, *RGS1* regulator of G protein signaling 1, *MMP9* matrix metallopeptidase 9, *SLC2A3* solute carrier family 2 (facilitated glucose transporter) member 3, *YES* Yamaguchi sarcoma viral oncogene homologue, *FASLG* fas ligand (TNF superfamily, member 6), *GZMA* granzyme A (granzyme 1, cytotoxic T lymphocyte-associated serine esterase 3), *PRF1* perforin-1, *CREM* cAMP responsive element modulator, *EREG* epiregulin, *AREG* amphiregulin, *DUSP2* dual specificity phosphatase 2, *DAAM1* disheveled-associated activator of morphogenesis 1, *ORM1* orosomucoid 1, *ARG1* arginase 1, *PDGFD* platelet-derived growth factor D, *SIK1* salt-inducible kinase 1, *TPST1* tyrosylprotein sulfotransferase 1, *LAIR2* leukocyte-associated immunoglobulin-like receptor 2, *RUNX3* runt-related transcription factor 3, *TGFBR3* transforming growth factor beta receptor III, *MLC1* megalencephalic leukoencephalopathy with subcortical cysts 1, *GRB2* growth factor receptor-bound protein 2, *rpm* revolutions per minute, *PGLYRP1* peptidoglycan recognition protein 1, *TXLNA* taxilin alpha, *CSF2* colony stimulating factor 2, *CXCR* chemokine (C-X-C motif) receptor, *DDIT4* DNA-damage-inducible transcript 4, *NF*-*κB* nuclear factor-kappa B, *MCP*-*1* monocyte chemoattractant protein-1, *MR* mannose receptor, *AMAC*-*1* alternative macrophage activation-associated C-C chemokine-1, *PPARGC1A* peroxisome proliferator-activated receptor gamma coactivator 1 alpha, *PPARGC1B* peroxisome proliferator-activated receptor gamma coactivator 1 beta, *PPARG*/*PPARD* peroxisome proliferator-activated receptor gamma or delta, *AGER* receptor for advanced glycosylation end product-specific receptor, *NCF1* nicotinamide adenine dinucleotide phosphate-oxidase subunit p47phox, *NOX2* NADPH oxidase 1 subunit gp91 phox, *TRAF6* TNF receptor-associated factor 6, *CLC* Charcot-Leyden crystal galectin, *PARD6G* par-6 family cell polarity regulator gamma, *INSL6* insulin-like 6, *RET* ret proto-oncogene, *CPA2* carboxypeptidase A2, *CPS1* carbamoyl-phosphate synthase 1, *SGOL1* shugoshin-like 1

## Gene Expression Studies in PBMCs—Endurance Training

Peripheral blood mononuclear cells (PBMCs) are a subpopulation of leukocytes and consist of approximately 70 % T lymphocytes, 5–10 % B lymphocyte, 15 % monocytes, 10–15 % natural killer (NK) cells, and 0.5–1 % dendritic cells [54] and are often used to study the immune response in relation to atherosclerosis [18].

Ullum et al. were one of the first to publish a paper where gene expression in PBMCs in response to exercise was studied [21]. They measured gene expression of interleukin (IL) 1A, IL1B, IL6, and tumor necrosis factor alpha (TNF) before and after an ergometric bicycle exercise, but did not find any effect on gene expression after exercise. These results were largely supported by Moldoveanu et al. [25], Bernecker et al. [35], and Natelson et al. [22]. Ostrowski et al. concluded slightly differently when showing that PBMC gene expression of interleukin 1 receptor antagonist (IL1RN) and IL1B were upregulated after a marathon race [23]. Xiang et al. showed an upregulation of messenger ribonucleic acid (mRNA) levels 1 week after a marathon race for several genes, among them IL4, GATA binding protein 3 (GATA3), chemokine (C-C-motif) receptor (CCR)4, CCR3, and CCR2 [37]. A change in the Th1/Th2 ratio was observed from pre- to post-marathon as interferon gamma (IFNG)/IL4 ratio and T cell-specific T-box transcription factor T-bet (TBX21)/GATA3 ratio decreased. In agreement with Xiang el al. [37], Ulven et al. showed an upregulation of GATA3 mRNA expression in PBMCs after ergometric cycling [36]. mRNA expression of IL1B, chemokine (C-X-C motif) ligand 16 (CXCL16), IL8, prostaglandin-endoperoxide synthase 2 (PTGS2), and TBX21 were upregulated, while toll-like receptor 2 (TLR2) mRNA expression was downregulated after exercise. Li et al. found an increase in the mRNA gene expression of the anti-inflammatory cytokine IL13 after one hour tai chi [32].

Toll-like receptors (TLRs) play an important role in the immune system by recognizing and initiating an inflammatory response to dangerous molecules, possibly leading to the transcription of cytokines and chemokines [55, 56]. Nickel et al. investigated how a marathon race affected the expression of TLRs in lean subjects exercising regularly compared to lean subjects and obese subjects exercising less regularly [33]. They found differences in mRNA expression of TLR4 and TLR7 between the groups.

There is some evidence that a reduced level of nitric oxide (NO) or an elevated level of superoxide (O_2_^−^) increase the risk of cardiovascular disease. The expression and activity of inducible nitric oxide synthase (NOS2) relative to endothelial nitric oxide synthase (NOS3) is important in the regulation of inflammation [31]. Jenkins et al. compared the mRNA expression of several genes related to the antioxidant defense system in sedentary and physically active males after a treadmill test [31]. In CD34^−^ PBMCs, NOS3 gene expression increased in the sedentary group compared to the active group after exercise. No change was seen in the expression level of NOS2 between groups. Niess et al. found an increase in the expression of NOS2 after a marathon race, but not after a graded treadmill test [26].

The abovementioned studies were performed using reverse transcription polymerase chain reaction (RT-PCR). When using RT-PCR, only a limited number of genes can be analyzed at the same time. With a whole genome transcriptomic approach, it is possible to analyze thousands of genes at the same time.

When using this approach, Kimsa et al. found that an acute bout of bicycling regulated several biological pathways such as cytokine-mediated signaling pathways (IL6, IL8, and IL1B), intracellular signaling (IL5RA, IL6, and IL8), cell communication and cell-to-cell signaling (interleukin 2 receptor beta (IL2RB), colony-stimulating factor 2 (CSF2), and interleukin 1 receptor-like 1 (IL1R1). In total, ten inflammation-related genes were changed after exercise [44].

Connolly et al. showed that 311 genes were altered in PBMCs from baseline to immediately after a cycle ergometer workout, 552 genes were regulated from end of exercise and 60 min into recovery while 292 genes were regulated between baseline and 60 min into recovery [38]. The majority of the genes upregulated from baseline to end of exercise were related to inflammation and stress. From end of exercise and 60 min into recovery, an upregulation of nuclear receptor subfamily 4, group A, member 2 (NR4A2) and regulator of G protein signaling 1 (RGS1) were observed. The mRNA levels of IL6 and IL10 were not affected by the exercise at any time point.

Radom-Aizik et al. investigated how a bout of ergometric cycling altered gene expression in PBMCs in early and late puberty females [13] and males [12]. They observed that there were differences in gene expression between genders and pubertal phase. Genes commonly regulated by exercise in all groups were related to growth, apoptosis, inflammation, and tissue repair.

Transforming growth factor beta (TGFB) is involved in proliferation and differentiation. Kimsa et al. identified 14 genes, related to the TGFB-signaling pathway, that were differently regulated in PBMCs after exercise [41]. Only runt-related transcription factor 3 (RUNX3), transforming growth factor, beta receptor III (TGFBR3), megalencephalic leukoencephalopathy with subcortical cysts 1 (MLC1), and growth factor receptor-bound protein 2 (GRB2) were significantly altered.

Even though these studies are differently designed and the exercise programs are of different duration and intensities, exercise seems to have an influence on PBMCs. Genes regulated in these studies are associated with stress, inflammation, and tissue repair.

## Gene Expression Studies in Leukocytes—Endurance Training

Heat shock proteins (HSP) have important functions as molecular chaperons and are produced in response to different stressful stimulus. It has also been shown that HSPs are able to function as powerful cytokines [57] by binding to TLR2 and TLR4 [51, 57].

Fehrenbach et al., Maltseva et al., and Sakharov et al. investigated how exercise might affect gene expression of HSPs after exercise. Fehrenbach et al. showed that mRNA expression of heat shock protein 70 kDa binding protein (HSPBP1) increased in athletes compared to untrained subjects after exercise [58], while Maltseva et al. found that running altered the gene expression of peptidoglycan recognition protein (PGLYRP1) and heat shock protein 70 kDa protein 1A (HSPA1A), but not of HSPBP1 [42]. Sakharov et al. found an increased gene expression of HSPA1A after a treadmill test [29].

Subjects already participating in leisure activities performed a strenuous treadmill exercise, followed by a moderate treadmill exercise two weeks later in a study performed by Buttner et al. [39]. The mRNA levels of several genes were upregulated in both types of exercise, but there were differences depending on training intensity. Upregulated genes belonged to pathways associated with inflammation, stress signaling, electrolyte and substrate transport, extracellular matrix, and transcription factors.

Training intensity was also studied by Sakharov et al. who showed that 310 genes were upregulated in skiers after performing an exhausting treadmill test (RTE), while 69 genes were upregulated in the same subjects after performing a moderate treadmill test (MT) [43]. Sixty-four of the genes were identical in the RTE and MT, indicating a greater change in gene expression in response to a strenuous exercise.

These studies show that an acute bout of exercise alters gene expression in leukocytes and that exercise intensity might influence the level of inflammatory markers. The HSP may also play an important role in the acute response to exercise, possibly by inducing TLRs [51].

## Gene Expression Studies in Monocytes and Lymphocytes—Endurance Training

Ferrer et al. wanted to assess the effects of swimming on the pro- and antioxidant system of lymphocytes [28]. They found an increase in gene expression of B cell CLL/lymphoma 2 (BCL2) and uncoupling protein (UCP)-3 after one hour swimming. No changes were seen in the expression of catalase, glutathione catalase (GPX), or peroxisome proliferator-activated receptor gamma, coactivator 1 alpha (PPARGC1A).

Heme oxygenase 1 (HO-1) is suggested to have both immune-protective and anti-inflammatory properties [27]. Thompson et al. performed a cross-over study were invited subjects ran for 75 min, and the following test day were sitting calmly in the laboratory. The HO-1 mRNA expression level increased after exercise, while no changes were seen when resting in the laboratory [27]. The results from Thompsons’ study [27] was confirmed by Sureda et al. who showed that HO-1 gene expression increased after moderate- and high-intensity endurance training in soccer players [30].

Thomas et al. investigated if exercise was associated with an activation of peroxisome proliferator-activated receptor gamma (PPARG) signaling in monocytes in response to an acute bout of exercise [34]. The mRNA expression level of thrombospondin receptor (CD36), nuclear receptor subfamily 1, group H, member 3 (NR1H3) and ATP-binding cassette, subfamily A, member 1 (ABCA1) were upregulated while the expression levels of PPARG, PPARGC1A, cholesteryl ester transfer protein (CETP), and lecithin-cholesterol acyltransferase1 (L-CAT) were unchanged. These genes are key regulators of lipid and energy metabolism, which also are closely linked to inflammation. Radom-Aizik et al. found that the mRNA levels of TNF, TLR4, and CD36 were downregulated after an acute bout of exercise in monocytes [45] contradicting the results of Thomas et al. [34].

In the studies mentioned above, several genes known to be involved, or related to the immune system, have been investigated, both in males and females, young and elderly, and at different exercise intensities. All factors appear to have an effect on the outcome measured [59]. Few studies have been performed investigating the effects of gender and age. Some more studies have been performed studying differences in training intensities, making Sakharov et al. hypothesize that passing the anaerobic threshold is responsible for the differences seen in gene expression in white blood cells between low- and high-intensity activities [43].

## Gene Expression Studies in PBMCs After Acute Resistance Exercise

Only two studies have been looking at gene expression in response to an acute bout of resistance exercise, both using a microarray approach. Carlson et al. recruited healthy men to perform 30 min of resistance training [40]. Several genes were regulated in response to the exercise, and the greatest transcriptional changes were seen in genes related to immune response, cellular communication, and matrix remodeling. These results are largely supported by Storey et al. who found that 202 genes, primarily involved in cell-to-cell signaling, immune cell trafficking, organism survival, cell cycle, and cell death, were regulated after strength exercise [46].

The study of Carlson et al. [40] and Storey et al. [46] indicate that also strength exercise gives an acute inflammatory response. Given the differences in muscle work between endurance and strength exercise, one might expect a different inflammatory response, although some of the same pathways appear to be regulated as indicated by these two studies.

## Prolonged Exercise and Gene Expression Studies

Table 2b summarizes the effects of prolonged exercise on gene expression in white blood cells from nine studies, seven of which were performed using PBMCs and two using leukocytes. In studies using strength exercise as the intervention (four studies), mRNA expression level was measured before and after an acute bout of exercise, both at baseline and after the intervention period. In the remaining studies, gene expression was measured once before and once after the training period.

## Gene Expression Studies in PBMCs and Leukocytes—Endurance Exercise

PBMC gene expression levels of IL6 and TNF in elite and recreational gymnasts were investigated by Tringali et al. [52]. The gene expression levels of IL6 and TNF were higher in elite gymnasts than in recreational gymnasts after half a year with exercise. Tringali et al. also showed that the IL6 gene expression level was higher in pre-pubertal girls than in girls having reached the menarche, and that the ratios of IL6/IL10 and TNF/IL10 were higher in elite gymnasts than in recreational gymnasts [52].

Eight weeks of a low-intensity training program elicited an increase in gene expression of PPARGC1A, peroxisome proliferator-activated receptor gamma, coactivator 1 beta (PPARGC1B), IL4, and CD 14 molecule (CD14) in leukocytes, while the mRNA expression levels of IL6, CXCL2, TNF, and CCL2 were downregulated in a study performed by Yakeu et al. [48].

Dias et al. used a microarray approach when discovering that 211 gene transcripts related to immune function, cell cycle processes, development, and growth were regulated in PBMCs after 18 weeks of endurance training. One hundred and fifty-two gene transcripts were upregulated while 59 were downregulated [53].

Thompson et al. took another perspective when they examined the effect of an exercise intervention followed by two weeks without training [15]. Fifty-three gene transcripts, primarily involved in cell cycling, cell-mediated immune response, and cell-to-cell signaling and interactions, were differently regulated after the exercise period. After a period of detraining, the expression levels of 22 of the 53 genes were unaltered while the expression of 31 genes returned to baseline.

Even though the abovementioned studies are performed using different subjects (male and female, young and elderly), the results indicate that regular and moderate endurance exercise seems to lower some of the pro-inflammatory markers and/or promote an anti-inflammatory profile in the body.

## Gene Expression Studies in PBMCs—Resistance Exercise

Jiménez-Jiménez et al. investigated the inflammatory response in PBMCs in elderly men before and after an eccentric training program [47]. Following both the first and the second bout of exercise, an increase in gene expression of NOS2 and PTGS2 were observed. IL6 mRNA increased after the first bout of exercise only. An attenuation of the acute inflammatory response after the training period for NOS2 and PTGS2 following the second exercise bout were observed.

Fernandez-Gonzalo et al. investigated how gene expression levels of CD14 and TLR4 were regulated in response to a bout of eccentric exercise performed before and after a training program in men [49] and women [50]. The mRNA expression levels of TNF and TNF receptor-associated factor 6 (TRAF6) were also studied in males and females, respectively. The acute bout increased the gene expression of CD14 and TLR4 in both male and female. The gene expression levels of TNF in men and TRAF6 in females were also increased after the first bout of exercise. The training period did not influence the acute response of CD14 or TLR4 mRNA gene expression, but the mRNA levels of TNF and TRAF6 were attenuated in males and females, respectively, after the second bout of exercise compared to the first bout in trained subjects.

When investigating the gene expression of IL10 and TNF after a resistance exercise program, Rodriguez-Miguelez et al. found an increase in the gene expression level of IL10 in the training group compared to the control group [51]. The authors concluded that resistance exercise may represent an effective tool to lower the pro-inflammatory status through an increased IL10/TNF-ratio.

Gano et al. found that the mRNA expression levels of advanced glycosylation end product-specific receptor (AGER), neutrophil cytosolic factor 1 (NCF1), and CCL2 were downregulated after an exercise period, improving the inflammatory/oxidative gene expression profile after a training period [16].

In all the studies using strength exercise, an acute bout of exercise has been included both at baseline and after exercise. With this design, the investigators will get valuable information about how one subject reacts to an acute bout of exercise while being trained compared to being untrained. This might be a valuable approach to study the molecular adaptions to exercise.

There are few studies performed investigating the effects of prolonged exercise on white blood cells and inflammation. The studies performed indicate that both resistance and endurance exercise promote an anti-inflammatory environment, or attenuate the acute response seen after a period of regular exercise.

## Discussion

Epidemiological studies have shown that regular physical activity improves health. The molecular mechanisms behind the beneficial effects have however not been completely elucidated. A number of factors such as individual genetic variability, different exercise protocols, the heterogeneous nature of exercise itself, and other lifestyle factors influence the effects observed [5]. Furthermore, exercise exerts a number of effects on the body such as improving insulin sensitivity and lipid profile in addition to lowering blood pressure [5]. All these factors influence each other making it hard to understand the complex interactions.

Exercise causes damage to the muscle resulting in disarrangement in fiber structures, loss of fiber integrity, and leakage of muscle protein. Trying to restore homeostasis, several repair processes starts, involving inflammation, resolution, muscle repair, and finally regeneration. The balance between pro- and anti-inflammatory cytokines and other signaling molecules seems to be important for the outcome of the repair and regenerating process [60].

It has been recognized that the muscle is an endocrine organ being able to influence other organs such as the immune system [61]. The immune system is also directly involved in the cellular and molecular events in the muscle after exercise by recruitment of macrophages, neutrophils, and lymphocytes participating in the clearance of necrotic tissue and producing signaling molecules [60]. It is therefore plausible to believe that exercise may induce a response in PBMCs [24]. It is also possible that PBMCs are influenced by the exercise itself, since the immune system is directly involved in the repair processes of the muscle after exercise [36]. Studying how exercise affects PBMC gene expression to elucidate the molecular mechanisms of exercise is therefore highly relevant.

Exercise seems to induce responses both in the innate and in the adaptive immune system. It appears to influence both signaling molecules and transcription factors, as shown in many of the studies included in this review. The innate and the adaptive immune systems mutually affect each other, even though the first reaction most likely occurs in the innate immune system. The response to exercise seems to be closely regulated and both pro- and inflammatory cytokines and signaling molecules are released.

TLRs are important constituents of the innate immune system. They are located at the cell surface and may be stimulated by endogenous molecules that potentially arise during exercise, such as HSPs [57] and interleukins [56]. An activation of the TLR may further elicit an immune response resulting in an upregulation of pro-inflammatory cytokines and chemokines, involving the NF-κB and MAPK-pathways.

Nickel et al. [33], Ulven et al. [36], and Fernandez-Gonzalo et al. [49, 50] investigated the gene expression levels of TLRs after exercise. Their results are somewhat conflicting, but show that gene expression levels of TLRs are affected by exercise. The explanation for the different results might be due to the type of exercise performed, the training intensities, and the training status of the individuals—all shown to influence the inflammatory response [62].

NF-κB is a transcription factor regulating a large number of genes, not only related to immune response, but also to cell survival, differentiation, and proliferation. NF-κB is expressed in all cells and is involved in both the innate and the adaptive immune system [63]. The pro-inflammatory effects of NF-κB are well known, but there are also evidence indicating an effect of NF-κB signaling in the resolution of an inflammatory response [64]. There is some evidence that NF-κB play an important role in the development of atherosclerosis [65].

NF-κB signaling pathways may be triggered by several stimuli such as NOS2 [56], HO-1 [30], TLRs [63], HSPs [57], TRAF [66], and cytokines such as IL1B and TNF [63], all genes investigated in articles included in this review [16, 24, 26, 27, 29, 30, 47, 50]. Some of the results are conflicting, which may be due to the differences in training intensities.

NADPH oxidases play an important role in the innate immune system. NADPH oxidases are enzymes producing superoxide (O_2_^−^), which again may produce reactive free radicals (ROS), thus being a possible contributor to atherosclerosis. It is hypothesized that the NCF1 play a role in TNF signaling and that impairing its expression may lower the oxidative and inflammatory status [67]. Gano et al. showed that the gene expression levels of NCF1 and NADPH oxidase 1, subunit gp91 phox (NOX2) were downregulated in PBMCs after two months of brisk walking [16], supporting this hypothesis.

Superoxide dismutases (SODs) are important molecules in the body’s defense against O_2_^−^ and free radicals [68]. SODs are able to catalyze the O_2_^−^ radical into regular oxygen or hydrogen peroxide (H_2_O_2_) and further to H_2_O. Jenkins et al. investigated changes in mRNA levels of SODs in sedentary and endurance-trained athletes after a treadmill test [31], and found an increase in SOD1 and SOD2 gene expression in CD34^−^ cells after exercise in the sedentary group.

There are two main classes of lymphocytes that are important in the adaptive immune responses. B lymphocytes are primarily responsible for the antibody responses, while T lymphocytes are responsible for the cell-mediated immune responses [69].

T lymphocytes may be divided into subsets such as T helper 1 (Th1) cells or the humoral/antibody T helper 2 (Th2) cells. Th1 cells primarily produce pro-inflammatory cytokines, while Th2 cells primarily produce anti-inflammatory cytokines [70, 71]. These responses are well-documented, but they are not the only cytokine pattern possible [70, 72]. It seems like the Th1-Th2 decision is important for proper immune function [70] and that the NF-κB pathway is involved in the regulation of this differentiation [20, 63].

When comparing studies looking at the inflammatory response to exercise, it is important to compare subjects at the same physical level. Capomaccio et al. [73] and Fehrenbach et al. [24] found different expression levels of IL6 and heat shock protein 27 kDa-associated protein 1 (HSPBAP1) in highly trained athletes compared to lightly trained and untrained subjects at rest. Different baseline values may cause a different inflammatory response after exercising. Comparison among different cells types should also be done with caution as different cell types might respond differently to exercise [74].

There is also evidence indicating that the immune function and the response to exercise is altered with age [5, 75] and across puberty [12, 76]. Elderly people also seem to have a higher basal level of inflammatory markers than younger people [16, 51, 77].

## Concluding Remarks and Future Perspectives

The effects of exercise on the body are multitudinous. Even though the study designs, the groups included, and the types of exercise used in the studies included in this review varies, it seems reasonable to conclude that exercise has an effect on cells of the immune system. Genes regulated after exercise are involved in inflammation, cellular communication, signal transduction, cellular protection, growth, and repair.

Overall, these studies show that an acute bout of exercise induces an immediate pro-inflammatory response, but that changes also occur in some of the anti-inflammatory markers. Prolonged and regular physical activity seems to promote an anti-inflammatory environment or attenuating the acute response to exercise, possibly reducing the risk of developing inflammatory-related diseases such as atherosclerosis [14, 78].

More research, preferably long-term standardized mechanistic studies, is needed to understand the impact of the observed change in gene expression, both in the muscles and in other organs, and to elucidate the complex interaction between white blood cells and muscle inflammatory response.
